# Evaluation of tympanometric alterations in patients subject to general anesthesia with nitrous oxide

**DOI:** 10.1016/S1808-8694(15)31323-9

**Published:** 2015-10-20

**Authors:** Fernanda Mossumez Fernandes Teixeira, Shiro Tomita, Marco Antônio de Melo Tavares de Lima

**Affiliations:** ^1^ Resident physician, Master in Otorhinolaryngology, UFRJ; ^2^ Physician, Faculty Professor, Discipline of Otorhinolaryngology, Federal University of Rio de Janeiro, Ph.D. in Otorhinolaryngology, EPM; ^3^ Physician, Ph.D. in Otorhinolaryngology, EPM, Joint Professor, Discipline of Otorhinolaryngology, HUCFF-UFRJ. Federal University of Rio de Janeiro

**Keywords:** óxido nitroso, pressão intratimpânica, alterações timpanométricas, nitrous oxide, intratympanic pressure, tympanometric alterations

## Abstract

**N**itrous oxide is an inhaling gas that can increase intratympanic pressure during the anesthetic act and cause negative pressure after it is discontinued, mainly in patients with Eustachian tube dysfunction. These pressure variations may come up with clinical implications such as tympanic membrane rupture, ossicular system disarticulation, haemotympanum, barotraumas, prosthesis displacement stapaedotomy and tympanic graft lateralization after tympanoplasty, in addition to serous fluid entrance into the middle ear during the negative pressure phase. **Aim**: To evaluate the nitrous oxide influence on the middle ear pressure in a population without tube malfunction performing pre and postoperative tympanometry. **Study design**: Transversal cohort. **Material and Method**: A prospective study was carried out with Universitário Clementino Fraga Filho Hospital- UFRJ inpatients submitted to general anesthesia with the use of 50% nitrous oxide from April to June 2003. It was also evaluated whether the duration of surgery, associated anesthetics, presence of allergic rhinitis and nasal septal deviation could contribute to the onset of intratympanic pressure alteration. **Results**: The sample was made up of 50 patients and in almost half of them (48%), postoperative tympanometry alterations (type C curve) were found when comparing to preoperative tympanometric control (type A curve). Neither gender nor age interfered in the onset of postoperative tympanometry alterations, similarly to surgery duration. The associated volatile anesthetic type, nasal septal deviation and allergic rhinitis were not able to influence postoperative middle ear pressure. **Conclusion**: Nitrous oxide modifies intratympanic pressure during the anesthetic act and after its discontinuation.

## INTRODUCTION

Nitrous oxide (azote protoxide) is a gas with analgesic properties that increases middle ear pressure upon being absorbed. After its suspension, it may cause subatmospheric pressure, owing to its quick reabsorption1., 2., 3.. This fact occurs because when it is administered to a patient breathing room temperature air it is switched to nitrogen^4^. However, protoxide input speed is 34 times faster than the output of nitrogen in the body5., 6., 7., 8., 9.. Owing to this imbalance, there is increase in pressure of little complacent cavities, such as the middle ear, in addition to increase in volume of complacent cavities, such as intestinal loop, pulmonary or renal cysts, up to the point that balance is restored10., 11., 12..

In the elimination phase, when administration is interrupted, there is quick reabsorption caused by low blood solubility; thus, in this step, negative pressure generated by middle ear (ME) leads to removal of fluids from intratympanic cavity mucosa capillaries, which may cause effusion of liquids generating serous otitis media (SOM)^13^. The magnitude of increase in volume or pressure caused by the gas is influenced by partial pressure of the lungs, blood flow and duration of protoxide administration^14^.

Both increase of intratympanic pressure and negative pressure may bring damage to ME function, such as tympanic membrane (TM) rupture, disarticulation of ossicle chain, hemotympanum, barotrauma, displacement of stapedotomy prosthesis, and lateralization of tympanoplasties' grafts1., 2., 15., 16..

There are reports that state these pressure modifications and their consequences may affect most of the population with auditory tube dysfunction, owing to the inability the auditory tube has to ventilate the ear, hindering rebalance of atmospheric pressure1., 3., 11., 14..

## OBJECTIVES

The present study intended to:

• assess the influence of nitrous oxide in middle ear pressure in a population without affections of the auditory tube by conducting pre and post-operative tympanometry in patients submitted to surgical procedures under general anesthesia with the gas;
• describe the frequency and type of tympanometric affections found in the postoperative period of the studied population and to assess whether age, gender, duration of surgery, type of associated volatile anesthetics, presence of allergic rhinitis and nasal septum deviation may influence the onset of these affections.

## LITERATURE REVIEW

### Nitrous oxide

Protoxide is used in general anesthesia as an adjuvant for the other inhaling anesthetic agents, acting as a carrier to other volatile agents, reducing their concentration4., 12.. This synergism allows reduction of consumption of agents, reducing recovery time^15^. It also has low hypnotic power and does not produce muscle relaxing effect, even though it is a potent analgesic agent^4^. Its inhalation may be used to treat pain, but use in chronic treatment is limited by side effects^12^.

Protoxide has the advantage of producing quick anesthetic induction and recovery, being considered one of the safest gases routinely used in daily practice15., 16.. It is quickly absorbed through lung alveoli, owing to its high diffusion capacity in the plasma. Most of it is excreted through the lungs within two to three minutes through expiration and only a small amount is excreted through the skin12., 15..

### Immittanciometry

Impedance is the opposition revealed by the sound conductive system of ME to the sound wave that penetrates into the external auditory canal (EAC)17., 18.. Complacence is the expression of easiness or magnitude of the tympanic-ossicle system and it is assessed by quantifying the sound energy reflected by the TM^19^. In the electroacoustic bridge, dynamic complacence is measured in the point of maximum complacence of tympanogram. If it is a normal ear, this point will be found close to zero pressure decaPascal, or slightly negative^18^. Thus, acoustic impedance of an object is the resistance presented to the passage of a sound wave and it depends on characteristics of vibration of the object.

The first systematic study of acoustic impedance in humans was carried out by Tröger in 1930. In 1946, Metz clinically applied the method by employing a mechanical bridge for acoustic measurement, allowing complete determinations of impedance, including phase and absorption components^17^.

Impedance measurements provide many advantages, among which the fact that it is an objective method of integrity and function of hearing peripheral mechanism and it is possible to estimate quantitative changes, in addition to being simple and easy to perform20., 21..

Complete immittanciometry comprises tympanometry, measurement of static complacence and thresholds of stapedial reflex^19^. In clinical practice, measurements made with tympanometry are routinely used in audiological assessments and in investigations performed for documentation^15^.

Tympanometry is an indirect test of auditory function, capable of assessing static and dynamic air pressure17., 19., 21., because it measures the capacity the membrane has to reflect a sound introduced in the EAC, as a response to gradual pressure modifications in the same space^18^. It is performed using an air pump with devices, which are introduced in the external ear by a probe to measure permeability of tympanic-ossicle system to the passage of sound wave^21^.

Most electroacoustic bridges currently used measure the energy that is reflected by the tympanic-ossicle set at the TM plane, that is, it measures the inverse of reactance to rigidity (complacence) expressed in equivalent cm3^18^. Any modifications to pressure modify acoustic impedance^20^.

### The influence of nitrous oxide in intratympanic pressure

ME is a little complacent cavity because three out of its six walls are bony, and only the one formed by the TM has some elasticity. The auditory tube is a bony-fibrous-cartilaginous duct that has three muscles: palatine veli tensor and elevator and salpingopharyngeal muscles, and it communicates the tympanic cavity with the nasopharynx. It is responsible for leveling intratympanic pressure with atmospheric pressure, through movements of auditory tube ostium at the level of rhinopharynx, which is made by the contraction of palatine veli muscle. In other words, its opening is determined by active movements, and its is physiologically reached by swallowing and yawning. Ostium closure is a passive movement made by relaxation of the muscle^22^.

Drainage of secretions to the nasopharynx is made by one of two ways: the mucociliary system, and the active mechanisms of auditory tube opening22., 25.. A normal tube easily balances differences of pressure, through frequent opening with passage of small volume of air: 1 to 1.5 milliliter^22^. In anesthetized patients, equalization of pressure depends on passive opening of the tube. In normal functioning ears, pressure increase caused by protoxide is limited by this opening, which occurs when intratympanic pressure exceeds 250mm H_2_O11., 14., 26.. In tube dysfunction cases, owing to inflammations, infections or scars resulting from adenoidectomy, there is no spontaneous ventilation14., 25.. Waun et al. (1967) reported that positive pressure could be transmitted to the ME through the auditory tube in those patients with mask-controlled ventilation.

Changes of pressure in the ear can also be influenced by volume of tympanomastoid cavity, mucosa vascularization and permeability of gas in the tympanic cavity27., 28..

Mastoid, considering it is a gas reservoir, minimized variations of pressure when the amount of gas in the ME goes through changes. There is an inverse correlation between extension of pneumatized mastoid and pressure peak during anesthesia with nitrous oxide, and negative pressure is more frequently found in those that are poorly pneumatized^29^.

Diffusion of gases in the ME may also have repercussions in the inner ear, nausea and vomiting during anesthetic recovery; they are the most frequent in patients that present high negative pressure owing to irritation of vestibular system by oval window membrane pull2., 3., 8., 30., 31..

Increase in intratympanic pressure occurs in the beginning of anesthesia, quickly increasing at the proportion of 10mm H_2_O per minute, reaching a peak at about 30 minutes16., 32..

Upon interrupting the administration of protoxide, intratympanic pressure normally slowly decreases, initially at the rate of 1mm H_2_O per minute, reaching pre-anesthetic levels between 40 and 50 minutes3., 25.. In cases of auditory tube dysfunction, the pressure may continue to be high even 20 minutes after the end of nitrous oxide inhalation11., 25., because there is no pressure balance through the auditory tube1., 2., 3..

During awakening, there is occurrence of swallowing, responsible for tube opening, allowing rebalance with atmospheric pressure^1^. However, owing to quick reabsorption of anesthetic, there may be negative intratympanic pressure8., 10.. It could provide occurrence of transudate from the local mucosa, reducing the gas volume in the ear, and consequently, its pressure^22^.

TM rupture may occur when intratympanic pressure is on average higher than 250mm H_2_O, which may happen with the use of nitrous oxide in patients with narrowed tube5., 25.. When there is negative pressure, if the tube ostium remains closed, there is also the possibility of a rupture, which can be avoided through voluntary maneuvers designated to open the tube in the early postoperative period, such as coughing or yawning. Another cause is the presence of neotympanum, which makes the membrane more fragile and increases the risk of rupture under stress of oscillation between positive and negative pressure^1^.

As to tympanoplasties, displacement of graft is a common finding in otological surgery with protoxide8., 11., 13., 33., caused by changes in intratympanic pressure during and right after the anesthesia8., 16., 33., which may cause significant implications in stability of ME reconstituted components^11^. For this reason, its use in this type of surgery and in patients that were submitted to ossicle reconstruction is questionable.

Its exclusion is preferable, recommending oxygen combined with air as a vehicle for inhaling anesthetic agents during the anesthesia for conservative mastoidectomy, tympanoplasty and other ME surgeries11., 33..

Hearing losses attributed to use of nitrous oxide may be conductive or sensorineural. Conductive hearing loss may occur by movement or displacement of prostheses in patients previously submitted to stapedotomy14., 34., 35., 36., tympanoplasties and conservative mastoidectomies^36^. Since ossicles were fixed in the TM, increases or reductions in intratympanic pressure may destabilize it and cause functional characteristics of ossicle chain, reducing hearing acuity10., 36..

Protoxide cessation may cause negative pressure, causing short-term hearing loss25., 30., 35.. If the pressure is sustained, it may cause conductive hearing loss by hemotympanum, ossicle chain disarticulation, TM rupture and barotrauma14., 35..

Sensorineural loss may occur through the formation of perilymphatic fistula35., 37., which may originate from two sources: explosive or implosive. In the first one, there is increase in cerebrospinal fluid pressure, which is directly conducted to the perilymph, via cochlear aqueduct or inner auditory canal. In the second case, there is considerable increase or intratympanic and auditory tube pressure^38^. The implosive force could create a rupture of the cochlear membrane and perilymphatic fistula in patients submitted to general anesthesia with the gas39., 40.. Thus, oval window rupture by implosive mechanism could occur in the anesthetic induction by a sudden movement of the TM, and consequently, a stapedial movement^37^.

## PATIENTS AND METHOD

### Design

We carried out a prospective study to assess the possible tympanometric affections in patients submitted to general anesthesia with nitrous oxide. The Ethics Committee for scientific study analysis at HUCFF-RJ approved it under number 100/02.

### Patients

The sample was selected during four months (April to July 2003) totaling a number of 118 cases, excluding 39 for not having type A tympanometry, 25 for surgical suspension, and 4 that required postoperative ICU care. Thus, there were 50 patients in the study.

### Inclusion criteria

Age over 18 years, no history of otological problems, normal otoscopy, submitted to general anesthesia with nitrous oxide; type A tympanometry; surgical duration equal or longer than one hour.

### Exclusion criteria

Patients submitted to neurosurgeries, nasal and otological surgeries, need for postoperative ICU care.

### Method

The selection of candidates to the study was conducted with a surgical map. We collected clinical history (using an objective questionnaire) and carried out complete ENT physical examination with tympanometry in both ears. Participation was defined after the confirmation of the fact that the surgical procedure had been carried out with nitrous oxide. Control tympanometry was conducted preoperatively with the patient in bed, during the hospitalization for the surgical procedure, using a device brand Amplaid, model 750. We carried out the exam with the patient lying down, bed head raised at 30º to facilitate postoperative assessment when there were difficulties in the bed. We adopted the classification by Jerger (1972) for tympanometric curves.

According to the anesthesiology service protocol at HUCCF, patients were submitted to pre-anesthetic medication (benzodiazepine: Midazolam -15mg or Diazepan-10mg PO - one hour before the surgery); anesthetic induction (Sodium Thiopental: 5 to 7 mg/Kg; Atracurium: 0.5 mg/Kg; Succinilcolin: 0.3mg/Kg; Xylocaine at 1%: 1 mg/Kg); Orotracheal intubation; combined inhaling anesthesia (nitrous oxide at 50%/O_2_ at 50% with isoflurane or nitrous oxide at 50%/O2 at 50% with enflurane). At this stage of anesthesia, patients were randomly divided to the groups treated with isoflurane or enflurane with combined inhaling anesthetic.

Statistical analysis was carried out to check whether there were statistically significant differences in variation of tympanometric parameters between the group of patients submitted to general anesthesia with the use of nitrous oxide (pre and postoperative). The adopted criteria for significance was 5%. The statistical analysis was processed by the statistical software SAS® System.

## RESULTS

We studied 50 patients aged 18 to 76 years, mean age of 41.6 years, standard deviation of 14.7 and median of 41 years, with predominance (40%) of age range between 38 and 47 years.

Nearly half of the studied sample (48%) presented postoperative tympanometric curve affections, with patients presenting type C curve, compatible with negative ME pressure.

Out of the total of 24 patients that presented postoperative affections, 13 had both ears affected; in unilateral cases, we could detect symmetry between them.

Age did not interfere in the onset of postoperative tympanometric affections in the studied group, which was also the case for gender.

Isoflurane was the preferred volatile agent in 76% of the cases. However, we observed that the choice of volatile anesthetic did not influence the onset of tympanometric affections.

Surgical time ranged from 60 to 360 minutes in the studied sample. The mean was 144.6 minutes, with standard deviation of 67.4 and median of 120 minutes. We also assessed whether surgical duration would have any effect over the presence of postoperative tympanometric affections, but there were no statistically significant differences among the groups.

We assessed whether nasal septum deviation and presence of allergic rhinitis would have any influence over the presence of postoperative tympanometric affections. However, we did not find any significant correlation between the groups.

## DISCUSSION

We assessed whether nitrous oxide could cause postoperative tympanometric affections in a population without auditory tube dysfunction. Given that intraoperative intratympanic pressure has been well established in the literature, we did not want to assess it during the surgical procedure.

Our findings are different from the literature, which normally states that it is required to have tube dysfunction to find such postoperative affections. Negative pressure would be a consequence of high intratympanic pressure during the surgical procedure, which is normally observed in patients with tube dysfunction, because normal ears have passive opening of auditory tube when there is elevation of intratympanic pressure1., 2., 3., 11., 14..

Richards et al. (1982) found a pattern defined as pressure variation in all patients that received protoxide in their study. Initially, there was gradual increase of pressure after administration of gas, followed by quick decrease, with subsequent increase, suggesting that this cycle was related with passive opening when intratympanic pressure reached its maximum peak. Thus, pressure was reestablished and there was tube closure and a new cycle was initiated.

Man et al. (1985) studied these effects using three combinations of gases: 1) environment air + 33% oxygen, 2) oxygen at 100%, and 3) 67% nitrous oxide and 33% oxygen, during pre and postoperative periods. In this sample, we assessed patients over the age of 18 years and without previous history of otological disease. As a result, in the first and second cases there were few pressure affections, differently from the third case, in which the pressure was quickly elevated.

In our sample, tympanometry was compatible with preoperative normal tube function (type A curve). However, in almost half of the patients (48%) there were tympanometric affections suggestive of negative pressure in postoperative ME (type C curve), using protoxide at 50%. This fact made us think that even though intraoperative intratympanic pressure was not assessed, the protoxide used at 50% concentration would have been enough to change it. We suggested the conduction of studies to analyze the incidence of postoperative tympanometric affections with the use of higher concentrations.

Peacock (1976) performed tympanometry in 50 children submitted to general anesthesia. The exam was performed one day before and within 18 and 24 hours after surgery. In 55% of the cases, there was reduction of ME complacence postoperatively, and in all of them the function was restored to normal levels within fewer than 6 days. There were no differences in intratympanic pressure in those ventilated with mask, when compared to those with orotracheal intubation, suggesting that positive pressure by ventilation-controlled mask does not interfere in the pressure during the surgical procedure. However, the sample comprised only children and it is known that the mastoid is little pneumatized in such age range, reason why there is higher intratympanic pressure. We did not perform late postoperative tympanometry; thus, we could not assess whether the negative pressure we found within the first 24 hours postoperatively persisted later, and if so, for how many days.

In our study, the minimum duration of studied surgical procedures was one hour, given that many authors reported that the necessary time for intratympanic pressure to reach its maximum point is about 30 to 40 minutes8., 17., 32.. Casey and Drake-Lee (1982) observed that this increase in intratympanic pressure during anesthesia with prototype is variable, but it does not exceed 10mmH_2_O/min. In this study, the maximum pressure found was after 30 to 40 minutes from the beginning of the anesthesia. Rasmussen (1967) reported initial increase of pressure of 20-100 mm/H_2_O per minute during anesthesia with nitrous oxide at 50%. As described by Ohrin (1985), pressure affections are geometrically varied, that is, if nitrous oxide was used at 50%, cavities of the body may have duplicated in size, whereas if the concentration was 75%, they may have quadruplicated in size.

TM rupture has been reported with the use of nitrous oxide. Waun et al. (1967) observed spontaneous rupture during administration of nitrous oxide in a patient with auditory tube dysfunction. Matz et al. (1967), in an experimental study with cats, tried to assess the rupture of TM following pressure increase with concentration at 66%. In this study, there was no TM rupture, probably owing to auditory tube opening. We did not find any case of TM rupture in our sample. To study it further, it would be necessary to have a significant increase in intratympanic pressure2., 5., 25..

Some cases of hearing loss have been attributed to use of nitrous oxide in general anesthesia. Coe (1987) reported that 57 patients with stapedial prostheses in situ were examined, showing audiometric evidence of conductive hearing loss after having been submitted to the gas. Journeaux et al. (1990) and Pau et al. (2000) reported cases of sensorineural loss after non-otological surgeries in which it was used. Waun et al. (1967) reported that the hearing drop could be in fact more frequent than reported by the patients. which may still be sedated within the first 24 to 48 hours after the surgery, not capable of observing this fact.

In our study, in the group of patients that presented postoperative tympanometric affections only one patient reported mild unilateral hearing acuity decrease, followed by ear fullness on the right side, but otoscopy was within the normal range and tympanogram was type C. It was not possible to perform audiometry because the patient had been submitted to abdominal surgery and could not be taken to the sound-proof booth.

Nitrous oxide is normally used associated with other volatile anesthetic agents, and there are many studies comparing ME pressure affections and the use of nitrous oxide and halogen agents27., 36.. To try to define whether some inhaling anesthetic agents used in combination could have some kind of postoperative influence, two inhaling agents, isoflurane and enflurane, were studied and we concluded that in none of the groups there were postoperative tympanometric affections.

Rasmussen (1967) did not find intratympanic pressure increase in patients treated with isoflurane. Katayama et al. (1992) noticed this variation and whether there were differences in behavior when protoxide was associated with isoflurane or enflurane, and they did not find any difference in pressure.

We tried to assess whether nasal septum deviation would play any influence in the occurrence of postoperative tympanometric affections. We studied whether the affected ear (right/left) would be related with homolateral septum deviation. This was thought because anatomical affections, such as septal malformation, could selectively affect drainage and the protective functions of ME^22^. However, we did not find any evidence of this association. In the studied literature, there were no references to this type of analysis.

Allergic rhinopathies affect the physiology of auditory tube. Allergic reaction (presence of mast cells) and hair affection may hinder rhythmic action of mucociliary complex, consequently hindering drainage functions, which are one of the factors that take to tube dysfunction^22^. For this reason, we assessed whether the patients with allergic rhinitis would have higher incidence of tympanometric affections. We did not observe any association in this case. In the studied literature, we did not find any references to this combination.

Given that nitrous oxide is potentially harmful to the hearing of susceptible subjects, specific issues such as previous otological surgery or ear-related symptom have to be investigated before the surgery9., 25., 26.. Previous history of ME surgery increases the risk of complications, in addition to the fact that absence of preoperative symptoms does not eliminate the risks of TM damage2., 9..

Patients submitted to ear surgery with reconstruction of ossicle chain are more vulnerable to damage as a result of pressure differential. Even though cases of oval and round window rupture may not be predicted with the use of nitrous oxide, it is certainly possible to avoid cases of ossicle displacement and damage to ME in patients who had been submitted to otological surgeries in the past^32^. Therefore, anesthesia with nitrous oxide should be avoided in these patients, if possible2., 8., 13..

Given that there was a high frequency of postoperative tympanometric abnormalities in our study, we suggest that during pre-anesthetic visits some questions about hearing problems should be included so as to select patients susceptible to the use of nitrous oxide, considering the existing alternatives^25^. In those patients with previous history of otological surgeries and auditory tube dysfunction we suggest that a preoperative assessment be performed by the ENT physician before elective surgeries under general anesthesia. To that end, it would be interesting to make clinicians and cardiologists aware of the importance of assessing these cases before the surgery.

## CONCLUSION

Nitrous oxide changes the pressure in the middle ear both in the induction phase and in the anesthetic resolution phase; duration of surgery did not influence the onset of postoperative tympanometric affections; we did not detect significant association between volatile anesthetics used combined with nitrous oxide and some influence on postoperative middle ear pressure; we did not find association between anatomical affections, such as nasal septum deviation and physiological abnormalities, such as presence of allergic rhinitis and onset of postoperative tympanometric affections.
